# Determinants of change in body weight and body fat distribution over 5.5 years in a sample of free-living black South African women

**DOI:** 10.5830/CVJA-2016-038

**Published:** 2016

**Authors:** Sarah Chantler, Kasha Dickie, Lisa K Micklesfield, Julia H Goedecke, Julia H Goedecke, Lisa K Micklesfield

**Affiliations:** Division of Exercise Science and Sports Medicine, Department of Human Biology, University of Cape Town, Cape Town, South Africa; Division of Exercise Science and Sports Medicine, Department of Human Biology, University of Cape Town, Cape Town, South Africa; Division of Exercise Science and Sports Medicine, Department of Human Biology, University of Cape Town, Cape Town, South Africa; Division of Exercise Science and Sports Medicine, Department of Human Biology, University of Cape Town, Cape Town, South Africa; Non-Communicable Disease Research Unit, South African Medical Research Council, Cape Town, South Africa; MRC/Wits Developmental Pathways for Health Research Unit, Department of Paediatrics, Faculty of Health Sciences, University of Witwatersrand, Johannesburg, South Africa

**Keywords:** body fat distribution, weight gain, black women, South Africa

## Abstract

**Objective:**

To identify socio-demographic and lifestyle determinants of weight gain in a sample of premenopasual black South African (SA) women.

**Methods:**

Changes in body composition (dual-energy X-ray absorptiometry, computerised tomography), socio-economic status (SES) and behavioural/lifestyle factors were measured in 64 black SA women at baseline (27 ± 8 years) and after 5.5 years.

**Results:**

A lower body mass index (BMI) and nulliparity, together with access to sanitation, were significant determinants of weight gain and change in body fat distribution over 5.5 years. In addition, younger women increased their body weight more than their older counterparts, but this association was not independent of other determinants.

**Conclusion:**

Further research is required to examine the effect of changing SES, as well as the full impact of childbearing on weight gain over time in younger women with lower BMIs. This information will suggest areas for possible intervention to prevent long-term weight gain in these women.

## Objective

Obesity and its co-morbidities continue to increase worldwide, with women from low- and middle-income countries (LMICs) being most affected.^1^ Within South Africa, the prevalence of overweight/obesity has increased from 56.2 to 64.8% in the most recent South African National Health and Nutrition Examination survey (SANHANES),^1^,^2^ confirming the problem within South Africa.

Our study in a cohort of black South African (SA) women reported a 9% increase in body weight over a 5.5-year period.^3^ The weight gain was attributed predominantly to an increase in fat mass, which was greatest in central compared to peripheral depots. The relative redistribution of body fat was associated with increases in fasting plasma glucose and triglyceride concentrations, with reduced insulin sensitivity and a compensatory increased insulin secretion at follow up.^3^

Other longitudinal studies measuring changes in body composition over time reported weight gain that ranged from 0.5–0.9 kg/year.^4^-^7^ However, these studies were undertaken in high-income countries (HICs). These data are valuable since to the authors’ knowledge, there are no known longitudinal studies from populations living in LMICs.

Determinants of weight gain in these studies include non-modifiable factors such as age, gender and race, and modifiable factors such as baseline body mass index (BMI),^5^ dietary intake,^4^,^7^-^12^ physical activity,^10^ socio-economic status (SES)^11^,^12^ and parity.13-9 Associations between these factors are often complex in nature, and it is difficult to draw conclusions regarding the relative contribution of these factors to increasing body weight in different populations. Furthermore, many of these studies failed to assess the impact of these determinants on changes in body composition or body fat distribution. Due to the risk of cardiometabolic disease associated with increasing total and central fat mass,^16^-^18^ and the possible protective benefits associated with peripheral fat mass,^19^ a greater understanding of the determinants of body composition changes are important to inform future intervention studies.

In addition, within LMICs, other factors such as urbanisation and the ‘transition’ from a traditional to a more westernised lifestyle have been associated with obesity and other non-communicable diseases.^20^ The relationship between SES and obesity in LMICs differs from that in HICs, with studies in LMICs reporting a positive association between SES and BMI, with the inverse association being reported in HICs.^21^ To our knowledge no longitudinal studies have assessed the impact of change in SES on body composition and body fat distribution.

Therefore, the aim of this study was to assess lifestyle factors and SES variables at baseline and the changes in these factors over a 5.5-year follow-up period, and how these are associated with changes in body weight and whole-body fat distribution in a sample of peri-urban free-living black SA women.

## Methods

Participants included a sample of 64 women from the original convenience sample of 240 apparently healthy premenopausal black SA women who were tested in 2005/06,^22^ and were followed up approximately 5.5 years later, as previously described.^3^ The original cohort of women were recruited at baseline from church groups, community centres, universities and through the local press, and were included in the study if they were (1) 18–45 years old; (2) had no known diseases and were not taking medication for type 2 diabetes (T2D), hypertension, HIV/AIDS, or any other metabolic diseases; (3) were not pregnant, lactating or postmenopausal (self-reported); and (4) were of SA ancestry (self-reported). At follow up, the original cohort of 240 women were contacted and invited to participate in the longitudinal follow-up study in 2010/11.

Testing procedures at baseline included body composition measures, questionnaires on SES and reproductive health, and an assessment of baseline physical activity and dietary intake. The dietary and physical activity assessment was not included at follow up. At follow-up testing, voluntary HIV screening was included. Participants were excluded on the basis of a confirmed positive HIV test (Sanitests Home Test Kits, SA). For ethical reasons, those who declined HIV screening were not excluded from the study.

The study was approved by the Human Research Ethics Committee of the Faculty of Health Sciences of the University of Cape Town. Before participating in the study, procedures and risks were explained to the subjects, and written informed consent was obtained.

Body composition was assessed using basic anthropometry (weight, height and circumference), dual-energy X-ray absorptiometry (DXA) and computerised tomography (CT) scans. DXA was used to measure whole-body composition (Discovery-W®, software version 12.7.3.7; Hologic, Bedford, MA). In vivo precision (CV) was 0.7 and 1.67% for fat-free softtissue mass and fat mass, respectively. Percentage fat mass for the whole body was obtained and fat mass for the various regions of interest, including the trunk, limbs, android and gynoid regions, were derived using DXA cut-off lines positioned at anatomical markers, as previously described.^23^ CT was used to measure abdominal visceral adipose tissue (VAT) and superficial adipose tissue (SAT) areas (Toshiba X-press Helical Scanner®; Toshiba Medical Systems, Tokyo, Japan) in 43 women at baseline and follow up.

A Xhosa-speaking field worker administered the sociodemographic questionnaire at baseline and follow up. The questionnaire included measures of SES such as housing density, asset index, educational level, current employment and household sanitation. Housing density was defined as the number of persons in the household divided by the number of rooms. Asset index was based on 14 appliances/items, reflecting the individual and household wealth and resources. These included electricity in the home, ownership of a television, radio, motor vehicle, fridge, stove/oven, washing machine, telephone, video machine, microwave, computer, cellular telephone and paid television channels (MNET or DSTV). Level of education was described as those who had completed grade 12 (secondary school) or lower. Participants were categorised as employed (including students) or unemployed. Sanitation was described as access to running water or a flush toilet inside or outside the house.

Behavioural factors included self-reported indicators of current smoking status (smoker or non-smoker), alcohol consumption (non-drinker or drinker of any alcohol), and hormonal contraceptive use (none, oral or injectable). Parity was defined as those who had children at baseline or follow up, and those who had children during the follow-up period.

Physical activity was assessed at baseline using the global physical activity questionnaire (GPAQ).^24^ As walking was the most frequent activity, walking for travel was used as a proxy for physical activity. Dietary intake was determined using a quantitative food frequency questionnaire,^25^ which has been validated in black SA women.^26^ A higher diet quality index – international (DQI-I) score represents a higher quality of dietary intake.

## Statistical analysis

Parametric data are presented as means and standard deviations and non-parametric data are presented as medians and interquartile ranges (IQR) and compared using paried t-tests and the Mann–Whitney U-test. Socio-demographic or categorical data are presented as percentages and were compared over the follow-up period using McNemar chi-squared tests. For univariate analysis, Spearman’s rank correlations were used to assess non-parametric associations between continuous variables (housing density, asset index) and the changes in body composition, while ANOVA was used to explore the effects of categorical variables (parity, access to sanitation, smoking, alcohol and walking for travel) on changes in body composition.

To analyse the effect of baseline age and BMI on changes in body composition, median age and accepted BMI classifications were used to create groups, and a two-way analysis of covariance (ANCOVA), adjusting for age, was performed. Based on the significant univariate associations with changes in body weight and body composition (baseline age and BMI, access to sanitation, parity, level of education and employment status, and changes in these SES and lifestyle variables), multiple stepwise linear regression was used to determine the independent contribution of these variables to changes in weight gain and body fat distribution over the 5.5-year follow-up period. Statistical significance was set at p < 0.05. Data were analysed using STATISTICA version 10 (Statsoft Inc. Tulsa, OK) and STATA 12.1 (StataCorp, College Station TX).

## Results

Subject characteristics, including body composition, SES and lifestyle variables at baseline and follow up are presented in Table 1. Mean percentage weight gain over the follow-up period was 8.8%, with an average increase of 1.2 kg/year. There was a significant increase in fat mass (16.4 ± 26.9%, p < 0.001), but no significant increase in fat-free soft-tissue mass (p = 0.234). The increase in fat mass was largely attributed to an increase in central fat mass, characterised by increases in trunk (as a percentage of total fat mass) and android fat mass, as well as both VAT and SAT areas. Conversely, there was a decrease in peripheral fat mass (gynoid and leg fat mass as a percentage of total fat mass).

**Table 1:** Socio-economic and lifestyle variables at baseline and after 5.5 years of follow up Indicator Baseline Follow

*Indicator*	*Baseline*	*Follow up*	*p-value*
Age (years)	27 ± 7.5	32 ± 7.6	–
Body composition			
Height (m)	1.6 ± 0.1	-	-
Weight (kg)	86.9 ± 19.6	92.8 ± 18.9	< 0.001
BMI (kg/m^2^)	33.8 ± 7.5	36.4 ± 7.7	< 0.001
Fat-free soft-tissue mass (kg)	45.6 ± 6.8	46.2 ± 6.3	0.234
Fat mass (kg)	36.3 ± 10.3	40.9 ± 10.6	< 0.001
Body fat (%)	42.3 ± 7.8	44.9 ± 6.4	< 0.001
Trunk fat mass (% total FM)	43.6 ± 5.8	46.2 ± 5.3	< 0.001
Leg fat mass (% total FM)	42.6 ± 6.3	40.1 ± 6.1	< 0.001
Android fat mass (% total FM)	7.7 ± 1.6	8.4 ± 1.6	< 0.001
Gynoid fat mass (% total FM)	19.3 ± 2.7	18.5 ± 2.4	< 0.001
VAT (cm^3^)	59 (37–93)	75 (49–110)	0.038
SAT (cm^3^)	508 (324–611)	499 (352–604)	0.013
Socio-demographic variables			
Education and employment			
Obtained grade 12 (%)	32.8	42.1	0.134
Employed/students (%)	32.8	45.3	0.042
Reproductive health			
Hormonal contraceptive use (%)	46.8^*#^	34.3	0.201
Parity (≥ 1 child) (%)	57.8^*^	85.9	0.001
Housing			
Housing density (people/room)	1.33 ± 0.9	1.38 ± 1.19	0.630
Running water inside house (%)	26.5	37.5	< 0.001
Flush toilet inside house (%)	26.5	40.6	0.001
Asset index (%14)	42.1 ± 19.4	55.8 ± 17.3	< 0.001
Lifestyle variables			
Current smoker (%)	12.5^#^	15.6	< 0.001
Consume alcohol (%)	37.5	48.4	0.291

Data are represented as either mean ± standard deviations or medians (interquartile range), Continuous data were compared using Wilcoxon rank test or dependent t-test, frequencies were compared using McNemar chi-squared test, significance p < 0.05.

*Significant difference in age between groups at baseline

#Significant difference in BMI between groups at baseline.

FM, fat mass; VAT, visceral adipose tissue; SAT, subcutaneous adipose tissue.

The measures of SES of the participants increased over the follow-up period, as characterised by increases in asset index (p < 0.001), access to flush toilets and running water inside the house (p < 0.001), and an increase in the number of participants who were employed (p < 0.05). At baseline, 58% of the women had at least one child, and this increased to 86% at follow up (p = 0.001). During the follow-up period, 24 (38%) women had one child, and three women (5%) had two children. Hormonal contraceptive use did not change significantly over time. The proportion of women who smoked increased over the follow-up period, but the proportion of women who consumed alcohol did not change significantly.

At baseline, the median moderate- to vigorous-intensity physical activity of the women was 60 min/day, with 70% of women using walking as a mode of travel. At baseline, the majority of dietary energy (kcal) was derived from carbohydrates (52.2%), followed by fat (34.8%) and protein (12.4%). The median total DQI-I score was 54 (IQR: 47–60), with a median (IQR) variety score of 17 (15–20), adequacy score of 25 (20–32), moderation score of 6 (3–12) and balance score of 3 (2–4). None of the baseline physical activity or dietary factors contributed significantly to weight change during the follow-up period so were not included in any further statistical analyses.

**Fig. 1.**
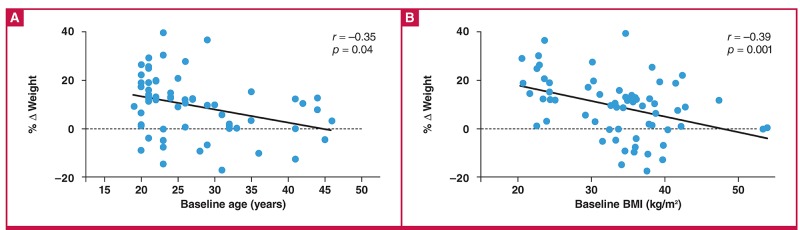
Relationship between baseline age, baseline BMI and relative change in body weight (%).

**Fig. 2.**
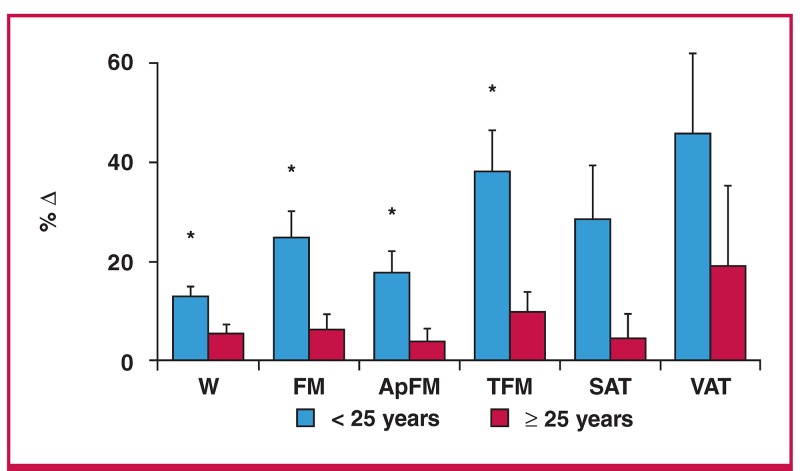
Percentage changes in body composition variables in younger (< 25 years) and older age groups (≥ 25 years). Data are means ± standard error. W, weight; FM, fat mass; ApFM, appendicular fat mass; TFM, trunk fat mass; SAT, superficial adipose tissue; VAT, visceral adipose tissue; *p < 0.01.

Although there was no association between age and BMI at baseline, both were inversly associated with change in body weight (Fig. 1A, B). To further investigate the effect of baseline age on changes in body composition, participants were divided into two categories, those above and those below the median age of 25 years (Fig. 2). The younger participants (< 25 years) gained significantly more body weight, total fat mass, appendicular fat mass, and trunk fat mass than the older participants (≥ 25 years) (p < 0.05), with a three-fold greater increase in fat mass in the younger compared to the older group (6.3 ± 6.9 vs 2.1 ± 6.5 kg, p = 0.016). The increase in fat mass in the younger versus the older group occurred mainly in the central (trunk) region (3.9 ± 3.7 vs 1.2 ± 3.4 kg, p = 0.005) rather than the appendicular region (2.4 ± 3.4 vs 0.8 ± 3.2 kg, p = 0.066).

To further explore the effect of baseline BMI on changes in body composition, the participants were separated into three BMI categories, non-obese (NO: BMI < 30 kg/m^2^, n = 17), obese class 1 (OBc1: BMI: 30–34.9 kg/m^2^, n = 17) and obese class 2 (OBc2: BMI ≥ 35 kg/m^2^, n = 35). The annual average weight gain was 1.8 ± 0.9, 1.2 ± 2.1 and 0.9 ± 1.9 kg in the NO, OBc1 and OBc2 groups, respectively.

**Fig. 3.**
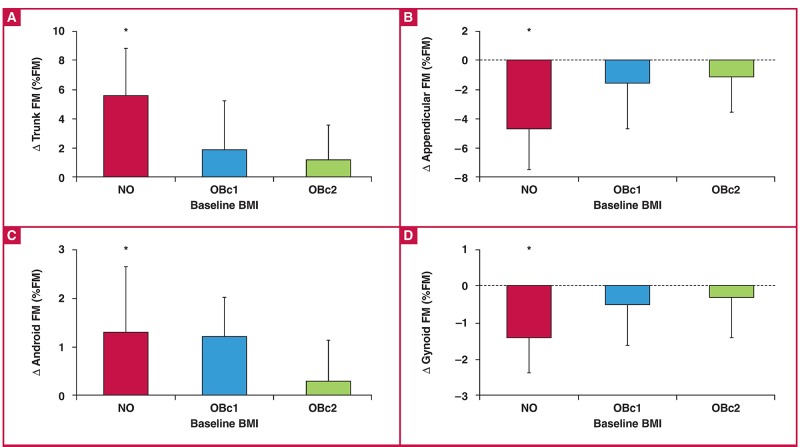
Changes in body fat distribution, expressed as a percentage of total fat mass (kg), in three baseline BMI groups. Data are represented as means ± standard deviation, FM; fat mass. Three BMI groups defined at baseline: NO:< 30 kg/m^2^, OBc1: 30–34.9 kg/m^2^ and OBc2: ≥ 35 kg/m^2^. *Change in NO group significantly different to both other BMI groups, p < 0.01 via Tukey post-hoc analysis.

Absolute and percentage changes in body composition over the 5.5-year follow-up period in the three BMI groups are presented in Table 2. There was no significant difference in age between the BMI groups (NO: 24.7 ± 1.8 vs OBc1: 28.7 ± 1.8 vs OBc2: 27.2 ± 1.3 years, p = 0.283). While the absolute changes (kg) in the various body composition variables were not significantly different between the groups, the percentage changes in body composition (relative to baseline) were significantly greater in the NO group compared to the other two groups. In addition, when expressed as a percentage of total fat mass, there were significant group × time interaction effects for the changes in body fat distribution, such that there was a significant increase in central fat mass (trunk and android as a percentage of fat mass) and decrease in peripheral fat mass (appendicular and gynoid as a percentage of fat mass) in the NO group, but not the two obese groups (Fig. 3).

**Table 2:** Changes in body composition over the 5.5-year follow-up period by baseline BMI groups

*Parameters*	*BMI*	*Baseline*	*Follow up*	*Absolute change*	*% change*	*p-value*
**	*(kg/m^2^)*	*(kg)*	*(kg)*	*(kg)*	**	*for % change*
Weight (kg)	NO	61.2 ± 9.2	71.7 ± 9.6	10.4 ± 5.4	17.6 ± 9.7#	0.009
	OBc1	85.9 ± 6.4	91.6 ± 12.2	5.6 ±12.1	6.8 ± 14.2	
	OBc2	100.4 ± 14.5	105.1 ± 15.2	4.7 ±10.5	5.2 ± 10.8	
Fat mass (kg)	NO	19.6 ± 5.8	26.3 ± 5.8	6.7 ± 4.1	39.6 ± 31.8^#^	< 0.001
	OBc1	37.5 ± 5.5	40.7 ± 9.4	2.5 ± 8.5	7.6 ± 23.8	
	OBc2	46.7 ± 10.2	50.0 ± 11.5	3.6 ± 7.2	8.0 ± 16.4	
TFM (kg)	NO	7.6 ± 2.7	11.6 ± 2.9	4.0 ± 2.1	63.2 ± 51.9^#^	< 0.001
	OBc1	16.5 ± 3.6	18.7 ± 5.3	1.8 ± 4.7	13.7 ± 32.9	
	OBc2	21.6 ± 4.9	23.7 ± 5.5	2.2 ± 3.9	11.0 ± 20.1	
ApFM (kg)	NO	11.1 ± 3.4	13.8 ± 3.2	2.6 ± 2.1	27.9 ± 26.1#	0.003
	OBc1	20.2 ± 3.2	21.2 ± 5.4	0.8 ± 4.0	3.3 ± 20.6	
	OBc2	24.1 ± 6.2	25.4 ± 7.0	1.4 ± 3.6	5.8 ± 15.3	
SAT (cm^2^)	NO	227.1 ± 100.5	300.4 ± 78.3	90.5 ± 68.4	58.9 ± 62.6^#^	0.033
	OBc1	487.7 ± 107.7	495.7 ± 107.7	15.6 ± 99.3	2.3 ± 27.2	
	OBc2	637.1 ± 118.9	651.6 ± 131.4	18.8 ± 84.1	2.2 ± 13.5	
VAT (cm^2^)	NO	31.9 ± 12.5	54.9 ± 29.4	24.8 ± 23.1	94.1 ± 101.1^#^	0.003
	OBc1	77.3 ± 44.3	81.4 ± 38.2	2.9 ± 38.9	15.4± 67.8	
	OBc2	73.4 ± 36.0	99.9 ± 38.6	1.5 ± 23.5	7.7± 30.5	

Data are represented as means ± standard deviations. Groups are divided into BMI at baseline as NO: < 30 kg/m2 (n = 17), OBc1: 30–34.9 kg/m2 (n = 17) and OBc2: ≥ 35 kg/m^2^ (n = 30).

^#^Change in NO group significantly greater than both OB groups, Kruskal–Wallis used for the relative change in SAT and VAT.

BMI, body mass index; TFM, trunk fat mass; ApFM, appendicular fat mass; SAT, subcutaneous adipose tissue, VAT, visceral adipose tissue.

As baseline BMI was a strong determinant of weight gain over the follow-up period, differences in baseline SES and lifestyle variables were also examined between BMI groups (NO, OBc1 and OBc2). There were no differences in baseline SES, reproductive health, moderate- to vigorous-intensity physical activity, walking for travel, absolute dietary intake or DQI-I between BMI groups (data not shown). Those who smoked at baseline had a significantly higher BMI than those who did not smoke.

Baseline housing density and asset index were not associated with change in body weight, body composition or body fat distribution. By contrast, other SES variables at baseline and the changes in these variables were associated with changes in body weight or changes in body composition over the follow-up period (Table 3). Baseline and changes in access to sanitation and employment had siginficant effects on weight gain over the 5.5 years, while education and contraceptive use did not.

**Table 3:** Change in body weight and trunk fat mass in response to differences in SES/behaviour/lifestyle variables

**	*Change in body weight (kg)*				
*SES/behaviour/lifestyle variable*	*n*	*Yes*	*n*	*No*	*p-value*
Access to inside running water at baseline?	16	1.7 ± 11.2	45	8.8 ± 8.8	0.012
Access to inside flush toilet at baseline?	16	2.3 ± 12.1	45	8.5 ± 8.8	0.032
Employed at baseline?	20	10.2 ± 10.9	41	5.1 ± 8.7	0.050
Grade 12 at baseline?	21	7.2 ± 8.8	40	6.5 ± 10.3	0.803
Hormonal contraceptive use at baseline?	30	8.0 ± 8.9	31	5.5 ± 10.6	0.982
Nulliparous at baseline?	25	10.7 ± 9.5	36	3.8 ± 8.9	0.005
Nulliparous at follow up?	9	16.6 ± 7.2	52	5.4 ± 9.3	0.001
Improvement in sanitation over time?	14	15.1 ± 7.5	44	4.8 ± 9.4	< 0.001
Loss of employment over time?	8	11.7 ± 6.4	53	5.8 ± 11.6	0.043
**	*Change in trunk fat mass (% TFM)*				
**	*n*	*Yes*	*n*	*No*	*p-value*
Improvement in level of education over time?	11	2.2 ± 3.3	53	4.6 ± 3.1	0.035
Nulliparous at baseline	25	3.7 ± 3.5	36	1.9 ± 3.1	0.044

Nulliparty had significant associations with changes in body weight as well as changes in body fat distribution. Parity was associated with redistribution of fat mass, with larger decreases in appendicular fat mass (percentage of total fat mass) (–3.1 ± 2.9 vs –1.5 ± 2.7%, p = 0.040) and gynoid fat mass (percentage of total fat mass) (–1.1 ± 1.0 vs –0.5 ± 1.2%, p = 0.088), and larger increases in trunk fat mass (percentage of total fat mass) (3.69 ± 3.5 vs 1.9 ± 3.1%, p = 0.044) and trunk:leg ratio (0.19 ± 0.2 vs 0.08 ± 0.1%, p = 0.004) in the nulliparous women compared to the women with children at baseline.

Furthermore, those women who were still nulliparous at follow up (n = 9) increased their body weight significantly more over the 5.5-year follow-up period than their childbearing counterparts (p = 0.001). There was a trend for those who had children over the 5.5-year period to increase their body weight less than those who already had children, but it was not significant after adjusting for baseline age and BMI. Those who increased their education level (n = 11) had a greater increase in relative trunk fat mass (percentage of fat mass) compared to those who did not (n = 53) (p = 0.035).

Dietary intake and physical activity at baseline, and baseline and follow-up smoking and alcohol intake were not associated with changes in body composition (data not shown).

Multiple regression analysis was used to explore the independent determinants of the changes in body weight and body fat distribution over the 5.5-year follow-up period (Table 4), based on the significant univariate analyses described above.

**Table 4:** Multivariate models for changes in body composition over the 5.5-year period

**	*Change in body weight (kg)*								
Variable	β			SEE			p-value		
Baseline BMI	–0.24			0.13			0.016		
Presence of running water and a flush toilet	–0.28			2.66			0.023		
Improvement in sanitation (toilet and water)	0.30			2.41			0.005		
Child/children at baseline	–0.42			2.07			0.000		
Children over follow-up period	–0.25			2.12			0.025		
R2 = 0.51, p < 0.001 VIF: 1.25.									
	*Change in body fat distribution*								
*Variable*	*Δ Trunk FM (% FM)*			*Δ Trunk:leg*			*Δ Gynoid FM (% FM)*		
**	*(R^2^ = 0.43) (p < 0.001)*			*(R^2^ = 0.35) (p < 0.001)*			*(R^2^ = 0.20 (p = 0.001)*		
*VIF*	*1.02*			*1.02*			*1.02*		
**	*β*	*SEE*	*p-value*	*β*	*SEE*	*p-value*	*β*	*SEE*	*p-value*
Baseline BMI	–0.61	0.04	< 0.001	–0.47	0.00	< 0.001	0.40	0.02	0.001
Child/children at baseline	–0.34	0.69	0.001	–0.42	0.03	< 0.001	0.22	0.27	0.022

Based on the regression model, increasing body weight over time was associated with a lower baseline BMI, being nulliparous at baseline, not having children during the follow-up period, lack of household sanitation at baseline and improved sanitation at follow up. This model explained 51% of the variance in the change in body weight (p < 0.001). The model that explained the greatest variance in the change in relative trunk fat mass (percentage of fat mass), change in trunk:leg ratio and change in relative gynoid fat mass (percentage of fat mass) (model B) included only baseline BMI and being nulliparous at baseline.

## Discussion

The main findings of this study were that lower BMI and nulliparity, together with sanitation as a proxy for SES, were significant determinants of weight gain and change in body fat distribution over a 5.5-year period in a sample of free-living periurban black SA women. In addition, younger women increased their body weight more than their older counterparts, but this association was not independent of baseline BMI.

The finding that there was an inverse relationship between baseline BMI and weight gain is similar to other studies in HICs. Few researchers have highlighted the effect of baseline BMI as a predictor of future weight gain. Brown et al.^5^ showed that a baseline BMI of 25–30 kg/m^2^, in conjunction with a high energy intake, was a significant determinant of weight gain over five years in middle-aged Australian women. Another study in the USA reported greater weight gain in those who were both younger and who had a lower baseline BMI.^4^ In addition, this study showed that a lower baseline BMI was associated with a greater redistribution of fat from the periphery to the central depots over time. The women with a lower BMI may have a higher capacity for increasing body weight and increased centralisation of fat mass over time. This highlights a group that is at increased risk and should be targeted for future interventions aimed at preventing an increase in body weight and centralisation of fat mass over time, due to the associated negative cardiometabolic outcomes.^18^

A younger age at baseline was also found to be associated with increased weight gain and centralisation of fat mass over the 5.5-year follow-up period, however this was not independent of baseline BMI. Women under 25 years of age increased their weight by an average of 1.2 kg/year, compared to 0.3 kg/year in those who were older than 25 years. These marked age-related differences in weight gain have been reported previously, with increases in body weight being more pronounced in the younger compared to the older age groups over the same time period.^4^,^6^ These studies reported increases in body weight of 0.93, 0.73, 0.61 and 0.17 kg/year in participants from 21–35, 35–45, 39 and 59 years of age, respectively.^4^-^6^ The CARDIA study, which included black women of a similar age to our study, showed that women between the ages of 18 and 20 years at baseline increased their weight by an average of 1.2 kg/year, compared to 0.9 kg/year over 10 years in those who were 27–30 years at baseline.^28^

Although several studies have shown an association between a younger age and body weight gain, none have measured changes in body composition or body fat distribution over time. This study also showed a greater increase in trunk fat mass, and abdominal VAT and SAT areas in the younger age group, reflecting an increased centralisation of fat mass. However, when entered into the multiple regression analysis, baseline age was no longer significant in predicting weight gain and centralisation of body fat over the follow-up period.

In the regression model, it was also shown that having a child over the follow-up period was associated with less weight gain over the 5.5-year follow-up period. This finding is in contrast to previous research that has reported that child bearing is weightpromoting. 29 Rosenberg et al.^14^ have shown in a group of black women that the first child was associated with a 0.4 kg/m^2^ larger increase in BMI compared to those who had a second/additional child. In other studies from the USA, a higher energy intake^30^ and lower SES^31^ increased the risk of poor postpartum weight loss, while a study from Brazil found that high prepregnancy weight and higher gestational weight gain^32^ both increased obesity risk.

Although the women in this study were of a very low SES (Table 1), weight gain was found to be lower in women who gave birth during the study period compared to those who did not have children. It has been shown that with exclusive breastfeeding, postpartum weight loss may be improved,^33^ and even though exclusive breastfeeding rates in SA are considered low (1.4% of infants at six months), breastfeeding as part of mixed feeding is still popular^33^ and may contribute to lower weight gain postpartum in these women.

Furthermore, recent data from SANHANES-1 reported that one in three women (32.4%) experience hunger in the urban informal (peri-urban) environment.^2^ Unfortunately, since this study did not assess breastfeeding rates or household food insecurity, it is difficult to draw further conclusions as to the effect of these factors on postpartum weight loss. Nonetheless, given the poor SES of the study population, it is likely that more children introduced into the house may promote greater food insecurity, facilitating higher postpartum weight loss. Notably, those who did not have children at baseline were significantly younger than those who already had children, illustrating the possible co-linearity between parity and age. The younger women, who were also nulliparous, were also significantly less active, further confounding the effect of parity on weight gain.

Longitudinal studies to determine risk of future weight gain in high-income populations often use more static variables of education, employment or income as proxies for SES. To our knowledge, there are no longitudinal studies examining the impact of changes in SES, on body weight or obesity risk, which may be an important factor to consider in this highly mobile population.

Although, in this study there was a significant improvement in SES indicators over the follow-up period, specifically sanitation, household assets, level of education and rate of employment, it is still worth noting that less than 50% of the women had completed secondary level education, were formally employed, or came from households with running water and a toilet inside. In spite of the improvements in SES within this population, the traditional markers of SES, including level of education and employment, and the changes in these markers over time, were not independently associated with changes in body weight or body fat distribution.

However, women who had access to sanitation at baseline, representing a higher SES, had smaller weight gains over time. Conversely, those who improved their sanitation (and hence SES) over the study period had larger gains in body weight. This may suggest that with improving SES, women may increase their body weight, whereas if SES is stable, body weight might remain more stable. In high-income populations, it has been shown that SES is inversely associated with obesity, with a stronger relationship in women than men.^12^ Conversely, in LMICs, studies have shown a positive association between SES and BMI,^34^,^35^ while others have shown an increasing prevalence of obesity in the lower SES groups.^36^ Therefore, sanitation rather than the traditional measures of SES may better reflect the poor SES in this study.

Although this study did not find any associations with change in body weight and baseline dietary intake, DQI-I or physical activity, other large longitudinal studies have shown that different dietary patterns or physical activity levels have been associated with weight gain over time.^8^,^37^,^38^ The most recent data from SANHANES-1 and other studies also highlight poorer dietary diversity in the urban informal (peri-urban) environment compared to the urban formal areas,^2^ which may illustrate the interaction between SES and dietary quality. Therefore, even though diet was not found to directly influence the body composition changes in this study, dietary intake is likely to be influenced by the SES of the women.

Furthermore, although the food frequency questionnaire used in this study has been validated in black SA women, the lack of association with changes in body composition may be due to limitations with reporting of dietary intake and change over time. Considering the body of evidence from other studies showing the impact of change in dietary intake on increases in body weight, this would be a priority to assess further.

Lastly, this study was unable to assess household food security. Due to its interaction with both SES and dietary quality, this would be vital to include in future research.

Although most women met the physical activity guidelines (≥ 30 min moderate- to vigorous-intensity physical activity per day, American College of Sports Medicine), baseline physical activity was not associated with weight gain. Most of the physical activity was reported to be for transport, which is of low intensity and may not confer any reasonable effect on energy balance.^39^ As with dietary intake, the use of subjective measures of physical activity (GPAQ) may be limiting due to the misinterpretation of light- and moderate-intensity activity, leading to falsely elevated levels of reported daily physical activity.^40^

While the change in parity independently influenced weight gain, the use of hormonal contraceptives was not associated with a change in body weight or body fat distribution in this study. Previous studies have found significant increases in body weight and central fat mass with the use of hormonal contraceptives,^41^ but due to the lack of data regarding length of usage, it is difficult to correctly estimate the effect of the hormonal contraceptive in these women. In addition, SES and access to healthcare may influence the choice, as well as the consistency of contraceptive use in this group and dilute the possible impact on weight change.

A limitation of the study was that measurements were only taken at two time points, which may limit our interpretation of subtle changes within the time period. Therefore, acute or shortterm changes could not be measured. Even though the sample size was limited, the longitudinal nature of this study contributes to knowledge regarding the determinants of weight gain and their impact over time in a population at high risk of obesity and metabolic disease. Since only baseline dietary intake and physical activity were measured, this would be vital to follow up in future research studies.

## Conclusion

This study showed that lower BMI and nulliparity in the younger women were significant determinants of weight gain and centralisation of body fat over 5.5 years. In addition, although higher SES at baseline was associated with a smaller change in body weight, improvements in SES over the follow-up period were associated with greater weight gain. Many health programmes are targeted at women of reproductive age (e.g. family planning clinics). Accordingly, the introduction of weight-management interventions in these clinics is recommended to prevent and manage weight gain in these vulnerable young women, as well as future generations due to the intergenerational transfer of risk. Research to understand the relationship between alternative measures of SES, including sanitation and housing, and weight gain are required to guide future policy recommendations.
